# Chitosan and Chitosan Oligosaccharide Enhance Cryopreservation of Rabbit Testicular Tissue

**DOI:** 10.3390/biology15151301

**Published:** 2026-08-05

**Authors:** Yinlin Yao, Yuguo Wen, Zhen Zhu, Xiaotian Liu, Qinyao Shi, Tianhai Chen, Kai Kang, Jiang Wu

**Affiliations:** 1 College of Coastal Agricultural Sciences, Guangdong Ocean University, Zhanjiang 524088, China; 15913349792@stu.gdou.edu.cn (Y.Y.);; 2 Guangdong Engineering Research Center of Livestock and Poultry Bio-Breeding, Guangdong Ocean University, Zhanjiang 524088, China

**Keywords:** rabbit testicular tissue, cryopreservation, chitosan oligosaccharide, chitosan, histomorphology

## Abstract

Testicular tissue cryopreservation is a key strategy for preserving fertility in adult males, yet optimal cryoprotective protocols have yet to be established. This study evaluated the effects of varying concentrations of chitosan oligosaccharide (COS, 0–25%) and chitosan (CS, 0–25%) on rabbit testicular tissues. Right testes from 35 male New Zealand rabbits were assigned to COS groups and left testes to CS groups. Each group was further divided into seven subgroups: fresh control, 0% control, and 5%, 10%, 15%, 20%, and 25% treatment groups. After one month of slow freezing, tissues were assessed by histomorphological scoring (cell swelling, cell loss, basement membrane integrity, and seminiferous tubule architecture) and seminiferous tubule diameter measurement. The 20% COS group achieved the highest histomorphological score, significantly exceeding the 0% control and comparable to the fresh control. Among CS treatments, the 15% group yielded the best score, also significantly higher than the 0% control. All frozen groups exhibited significantly reduced seminiferous tubule diameters compared with the fresh control, and these diameter changes did not correlate with the morphological scores. Concentrations exceeding the optimal level led to lose tubular structure and severe exfoliation of epithelial cells, suggesting that excessive osmotic stress may have contributed to the observed structural damage. COS demonstrated superior cryoprotection compared with CS under the present experimental conditions, which may be attributed to its lower molecular weight and improved diffusibility. These findings indicate that COS may represent a promising candidate for optimizing polysaccharide-based cryoprotectants in testicular tissue banking, although further functional validation is required.

## Simple Summary

Freezing testicular tissue offers a way to preserve future fertility for boys and young men who face infertility risks from cancer therapy. However, the freezing and thawing process often damages tissue due to ice crystallization and osmotic stress. In this study, we tested whether adding two chitin-derived substances—chitosan and its smaller derivative, chitosan oligosaccharide—to the cryoprotective solution could better protect testicular tissue from such damage. We obtained testicular tissues from 35 adult male rabbits, exposed them to varying concentrations of each substance, cryopreserved the samples for one month, and then evaluated the histological structure post-thaw. Both substances provided protection, but only at specific concentrations: 15% for chitosan and 20% for chitosan oligosaccharide. Higher concentrations appeared detrimental, suggesting an optimal concentration threshold. Chitosan oligosaccharide outperformed chitosan overall, which may be related to its lower molecular weight and potentially more effective tissue penetration. These findings suggest that chitosan oligosaccharide is a promising additive for improving testicular tissue cryopreservation, which may ultimately have implications for fertility preservation in both adult and pediatric populations, as well as for safeguarding valuable animal genetic resources, although further validation in immature testicular tissues is warranted.

## 1. Introduction

Cryopreservation of testicular tissue is a vital strategy for fertility preservation, with applications in both human medicine and animal conservation. In human clinical practice, semen cryopreservation remains the standard and preferred option for most adult male patients. Testicular tissue cryopreservation is primarily indicated for prepubertal patients at risk of infertility following cancer therapy, as these patients cannot produce mature spermatozoa for conventional semen cryopreservation. For adult patients, this approach is generally reserved for selected cases, including those with obstructive azoospermia, severe oligozoospermia, or other conditions that prevent sperm collection or preservation [1,2,3]. In addition, this technology has significant value for the conservation of genetically valuable or endangered animals that die unexpectedly. Unlike semen cryopreservation, which is widely available for adult males, testicular tissue retains spermatogonial stem cells alongside their native microenvironment, which sustains complex crosstalk among Sertoli cells, Leydig cells, and peritubular myoid cells [4]. After thawing, spermatogenesis can be restored in testicular tissue via in vitro culture or xenotransplantation. In several animal models, these approaches have resulted in the production of functional sperm and viable offspring [5,6]. This technology constitutes an important platform for research in reproductive biology, the conservation of genetic material of endangered species, and the development of assisted reproductive technologies [5].

Cryopreservation of testicular tissue is generally more challenging than cryopreservation of semen or isolated cell suspensions. Testicular tissue contains germ and somatic cell populations that differ in membrane permeability, osmotic behavior, and susceptibility to cryoinjury [7]. During slow freezing, ice crystals first form in the extracellular compartment, elevating solute concentrations in unfrozen regions. Cells lose water in response to the hyperosmotic extracellular environment, leading to cellular dehydration, shrinkage, and osmotic stress that can compromise membrane integrity [8]. Insufficient dehydration leaves residual intracellular water, which can form lethal intracellular ice crystals upon rapid cooling. Early theories considered intracellular ice universally cytotoxic [9]; however, recent studies suggest that the relationship between intracellular ice formation and cell death is more complex, and that cell survival may depend on factors such as ice crystal size, location, and warming conditions, as well as the extent of recrystallization during thawing [10,11,12,13,14,15]. Moreover, oxidative stress generated throughout the freeze–thaw cycle further promotes cell apoptosis and disrupts tissue architecture. Accordingly, supplementation of cryopreservation media with effective, low-toxicity cytoprotective additives has emerged as an important strategy for improving cryopreservation outcomes [16].

Among cryoprotective additives, functional carbohydrates have been proven to effectively alleviate freeze–thaw injury to testicular tissue in previous studies, verifying their feasibility as cryoprotectant supplements [17,18,19]. Nevertheless, functional carbohydrates represent a wide variety of compounds exhibiting diverse molecular structures, physicochemical characteristics, and biological functions, and their protective roles in testicular tissue cryopreservation require further investigation. Chitosan (CS), a natural polysaccharide derived from the deacetylation of chitin, possesses favorable biocompatibility [20]. A recent study reported that supplementation with CS during cold storage of buck semen enhanced sperm motility and antioxidant capacity [21]. Among chitosan-derived compounds, COS, produced by enzymatic or acid hydrolysis of CS, is distinguished by its high water solubility and minimal cytotoxicity [22]. Notably, COS features superior water solubility and a low molecular weight, enabling it to penetrate deep into tissues to exert protective functions. To protect germ cells, COS has been demonstrated to suppress excessive reactive oxygen species accumulation, inhibit necroptosis, and restore mitochondrial function in spermatogonia [23]. Multi-omics analysis has revealed that COS activates the testicular PI3K-Akt signaling pathway [24]. In addition to its reported effects, this pathway is well known to regulate several other fundamental cellular processes, including inhibition of apoptosis, stimulation of cell proliferation, regulation of energy metabolism, and enhancement of cellular defenses against oxidative stress, thereby improving testicular development and spermatogenesis in mice [25]. Although both COS and CS have demonstrated protective potential for germ cells, research exploring their application in testicular tissue cryopreservation is still lacking. In particular, the optimal supplemental concentrations and corresponding cryoprotective efficacies of these two compounds for testicular tissue remain to be determined.

New Zealand rabbits were selected as the experimental model for this study due to their well-documented testicular anatomy and histological characteristics [26,27,28], as well as their widespread availability and reproductive physiology, which make them a suitable and reproducible model for testicular cryopreservation research. This study systematically evaluated the effects of serial concentrations (0–25%) of COS and CS on post-thaw testicular histomorphological scores (covering cell swelling, cell loss, basement membrane integrity, and seminiferous tubule architecture) as well as seminiferous tubule diameter. The objectives of this study were to determine the optimal concentration of each functional carbohydrate and to compare their cryoprotective efficacy. The findings are expected to provide theoretical support for testicular tissue cryopreservation and offer potential applications for germplasm conservation in rabbits and other animal species.

## 2. Materials and Methods

### 2.1. Experimental Animals

All animals with an average body weight of 2.5 ± 0.5 kg were housed in a specific-pathogen-free facility with free access to a commercial pelleted rabbit diet and fresh water, under a 12 h light/dark cycle, 22 ± 2 °C ambient temperature, and 50–60% relative humidity. All experimental procedures were approved by the Laboratory Animal Ethics Committee of Guangdong Ocean University (Approval No. GDOU-LAE-2024-003, 8 March 2024).

### 2.2. Samples Collection and Processing

Rabbits were sedated by intramuscular injection of xylazine (5 mg/kg) prior to euthanasia. Euthanasia was then performed via auricular vein injection with an overdose of pentobarbital sodium at a dose of 150 mg/kg. Death was confirmed by the absence of heartbeat and corneal reflex before tissue collection. Both testes were excised for tissue processing after death was confirmed. The tissues were rinsed repeatedly in pre-cooled phosphate-buffered saline (PBS) supplemented with 1% penicillin–streptomycin. Under 4 °C, the epididymis, tunica albuginea, rete testis, efferent ductules, fat pads, and other surrounding tissues were carefully removed using sterile forceps and ophthalmic scissors. The testes were cut into 1–2 mm^3^ cubes. A small amount of pre-cooled PBS was added intermittently to keep the tissues moist. To minimize inter-individual variations arising from genetic or physiological differences between animals, a paired-sample design was employed: the left testis from each rabbit was assigned to the CS treatment series, while the right testis from the same animal was assigned to the COS treatment series [28,29]. This approach ensures that any differences observed between the CS and COS groups are attributable to the additives themselves rather than to inter-individual variability. Within each treatment group (COS or CS), the testicular tissue fragments were randomly allocated to seven groups (*n* = 5 testes per group): a fresh control group (not cryopreserved), a cryopreserved group without COS or CS supplementation, and five cryopreserved treatment groups supplemented with 5%, 10%, 15%, 20%, and 25% of the respective additive (COS or CS). Testes assigned to the CS group were allocated using the same experimental design.

### 2.3. Preparation of Cryoprotective Solutions

The testicular tissue cubes were cryopreserved in Dulbecco’s modified Eagle’s medium (DMEM; Invitrogen, Carlsbad, CA, USA) supplemented with 10% fetal bovine serum (FBS; Hyclone, Logan, UT, USA), 10% dimethyl sulfoxide (DMSO; Sigma, St. Louis, MO, USA), 5% penicillin–streptomycin (500 U/mL; Hyclone), and the corresponding concentrations (0%, 5%, 10%, 15%, 20%, or 25%) of COS (chitosan oligosaccharide, molecular weight ≤ 1000, MACKLIN, Shanghai, China, cat. C875644) or CS (chitosan, deacetylation degree ≥ 90%, MACKLIN, Shanghai, China, cat. C909112). The detailed formulation is shown in Table 1.

### 2.4. Cryopreservation and Thawing of Testicular Tissues

The interval between sample collection and the initiation of cryopreservation was kept within 1 h. The cryovials containing testicular tissue cubes and cryoprotective solutions of corresponding concentrations were mixed thoroughly by vortexing. A slow freezing protocol was applied: the cryovials were equilibrated at 4 °C for 2 h, then at −20 °C for another 2 h, and finally stored at −80 °C for one month [19]. Five replicates were set for each treatment. After one month of cryopreservation, the cryovials were rapidly thawed in a water bath at 37 °C. The supernatant was discarded after centrifugation. Tissues were rinsed several times with pre-warmed 37 °C PBS, with centrifugation at 3000 rpm for 5 min between washes, to fully remove residual cryoprotectant. Treated tissues were collected for subsequent analyses.

### 2.5. Preparation of Testicular Tissue Sections and HE Staining

Thawed testicular tissues were fixed in 10% neutral buffered formalin for 24 h at room temperature. After fixation, tissues were dehydrated through a graded ethanol series (70%, 80%, 95%, and 100%, 1 h each), cleared in xylene (two changes, 30 min each), and embedded in paraffin wax using a tissue dehydrator (Servicebio, Wuhan, China, model ATD-405), paraffin embedding machine (Servicebio, Wuhan, China, model SWE-JBL5), and microtome (Servicebio, Wuhan, China, model PM-24). The embedded tissues were sectioned at 4 μm thickness and mounted on glass slides. For staining, sections were deparaffinized in xylene (two changes, 10 min each) and rehydrated through a descending ethanol series (100%, 95%, 80%, and 70%, 5 min each). Sections were then subjected to pretreatment with a pretreatment solution (Servicebio, Wuhan, China, cat. G1204) for 1 min, stained with hematoxylin (Servicebio, Wuhan, China, cat. G1076) for 3 min, rinsed in running tap water, differentiated in differentiation solution (Servicebio, Wuhan, China, cat. G1076) for 3–5 s, rinsed in tap water, treated with bluing solution (Servicebio, Wuhan, China, cat. G1076) for 3–6 s, and counterstained with eosin (Servicebio, Wuhan, China, cat. G1076) for 15 s after dehydration in 95% alcohol for 1 min [30]. Subsequently, sections were dehydrated through graded ethanol, cleared in n-butanol (Sinopharm Chemical Reagent Co., Ltd., Shanghai, China, cat. 100052190) and xylene (Shanghai Lingfeng Chemical Reagent Co., Ltd., Shanghai, China, cat. X100301-S5L), and mounted with neutral balsam (Sinopharm Chemical Reagent Co., Ltd., Shanghai, China, cat. 10004160). Ethanol was obtained from Sinopharm Chemical Reagent Co., Ltd. (Shanghai, China, cat. 100092683). Three sections were randomly selected from each treatment group. Five visual fields per section were observed and photographed under an optical microscope.

### 2.6. Histomorphological Scoring

HE-stained sections were evaluated based on five indicators: cell swelling, cell loss, basement membrane rupture, basement membrane shrinkage, and structural integrity of seminiferous tubules. Each indicator was scored on a scale of 0 to 2, where 0 represents the most severe damage, 1 represents moderate damage, and 2 represents normal or near-normal morphology. The total score was calculated as the sum of the five individual indicator scores, ranging from 0 to 10, with higher scores indicating better histological preservation (i.e., closer to the fresh control morphology). All sections were scored independently by three observers who were blinded to the treatment groups. Inter-observer agreement was assessed using Cohen‘s kappa coefficient, which ranged from 0.82 to 0.91 across the five parameters, indicating excellent consistency among the observers [31,32]. The average value of the three readings was adopted for further analysis.

### 2.7. Measurement of Seminiferous Tubule Diameter

Seminiferous tubule diameter was measured using ImageJ software (version 1.54p). Two perpendicular diameters (the longest and shortest axes) of each tubule were measured, and their average was taken as the final diameter.

### 2.8. Statistical Analysis

Prior to ANOVA, data normality was assessed using the Shapiro–Wilk test, and homogeneity of variances was verified using Levene’s test. The Shapiro–Wilk test confirmed that the data were normally distributed for all groups (*p* > 0.05), and Levene’s test confirmed homogeneity of variances (*p* > 0.05) for both COS and CS treatment series, supporting the use of parametric methods. Each treatment contained five biological replicates. Data were analyzed using SPSS 27.0 and presented as mean ± standard deviation (mean ± SD). One-way analysis of variance (One-way ANOVA) was performed separately for the COS group and CS group to detect significant differences among different concentrations. Duncan’s multiple range test was applied for post hoc comparisons when significant differences were observed. A difference of *p <* 0.05 was considered statistically significant. Graphs were plotted using GraphPad Prism 10.

## 3. Results

### 3.1. Morphological Observation of Thawed Testicular Tissues

**Figure 1:**
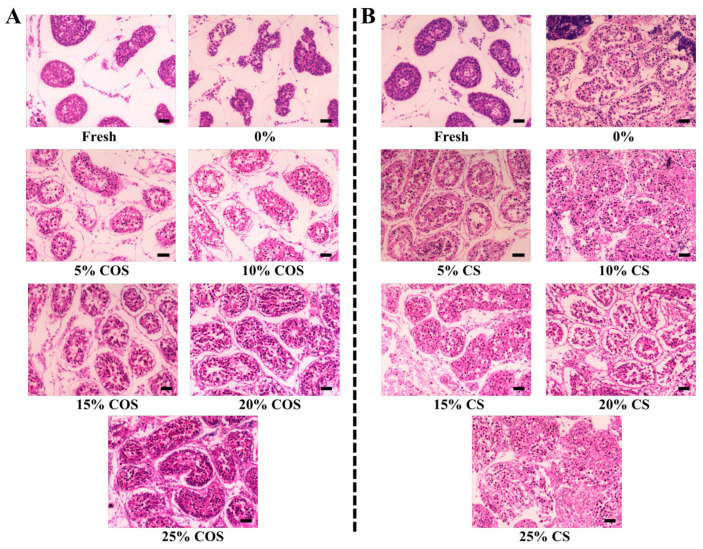
Morphological structure of rabbit testicular tissues in fresh control and groups treated with different concentrations of COS and CS (100×, scale bar=100 μm). (**A**) COS groups; (**B**) CS groups. HE staining: nuclei appear blue-purple, while cytoplasm and extracellular matrix appear red. COS, chitosan oligosaccharide; CS, chitosan.

In the fresh control group, the seminiferous tubules exhibited intact and sharply demarcated contours, with well-organized seminiferous epithelium (Figure 1). The seminiferous epithelium displayed tightly packed Sertoli cells and germ cells at all developmental stages; no obvious vacuolation or cellular shedding was observed. Interstitial tissue was normally distributed, indicating excellent viability of fresh tissues.

After cryopreservation and thawing, varying degrees of morphological alterations were detected across all treatment groups when compared with the fresh control, with the 0% additive group suffering the most severe injuries. Seminiferous tubules underwent severe atrophy and structural disintegration, accompanied by indistinct borders, while the seminiferous epithelium displayed severe disorganization and massive cellular shedding.

Among the cryopreserved groups, morphological preservation improved progressively with rising concentrations of COS or CS. Within the COS series, partial recovery of tubular outlines and alleviated cell shedding were observed at 5% and 10% concentrations, although the seminiferous epithelium remained disarranged. The 15% COS group largely retained intact tubular architecture and ordered epithelial layers, with markedly reduced cell loss and normally shaped nuclei in most cells. The 20% COS group achieved the optimal morphological preservation: fully intact tubular contours, compact and well-organized seminiferous epithelium, minimal vacuolation and cell shedding, uniform staining, and histological features highly comparable to the fresh control. At the maximum concentration of 25%, tubular outlines remained recognizable, but partial disorganization of cell arrangement emerged.

For the CS treatment series, compared with the 0% group, the 5% and 10% groups exhibited milder tubular structural damage, yet prominent cell shedding and widespread karyopyknosis persisted. The 15% CS group yielded the best outcomes, characterized by distinct tubular borders, orderly seminiferous epithelium, and significantly diminished cell loss. Morphological deterioration occurred in the 20% CS group, featuring partially distorted tubule outlines and aggravated cell shedding, while tissue damage was further exacerbated at 25% CS, manifesting as loose tubular structure and severe exfoliation of epithelial cells.

The above histological alterations were quantified via histomorphological scoring (Table 2 and Table 3). Among the COS groups, the 20% group attained the highest total score of 9.50 ± 0.32, which showed no statistically significant difference from the fresh control group (9.65 ± 0.67, *p* > 0.05) and was markedly higher than all other COS groups (*p <* 0.05). Within the CS series, the 15% group obtained the peak score of 8.18 ± 0.36, significantly exceeding the 0% control and all other CS groups (*p* < 0.05).

### 3.2. Analysis of Seminiferous Tubule Diameter

**Figure 2:**
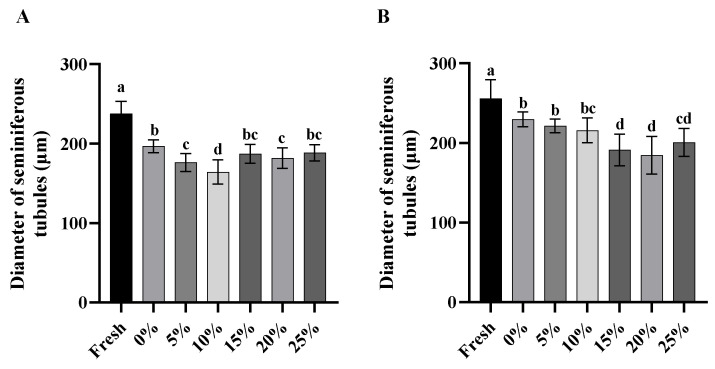
Effects of different concentrations of COS (**A**) and CS (**B**) on seminiferous tubule diameter of rabbit testicular tissues after cryopreservation. Different lowercase letters indicate significant differences (*p <* 0.05), while identical lowercase letters represent no significant difference (*p >* 0.05). COS, chitosan oligosaccharide; CS, chitosan.

Results of seminiferous tubule diameter measurement are presented in Figure 2. Within the COS groups, the fresh control group exhibited the largest seminiferous tubule diameter (237.78 μm), and all frozen groups showed significantly smaller tubule diameters relative to the fresh control (*p* < 0.05). Compared with the 0% group (196.94 μm), tubule diameters did not show an obvious recovery trend across 5–25% COS groups.

In the CS groups, the fresh control group had the maximum seminiferous tubule diameter (255.38 μm), and all frozen groups possessed markedly smaller diameters than the fresh control (*p* < 0.05). Tubule diameters displayed a continuous downward trend from 5% to 25% CS when compared with the 0% group (229.83 μm), with no sign of recovery toward the fresh control value. The 5% CS group yielded the largest diameter (221.55 μm), which was closest to that of the 0% group. Diameters gradually decreased at 10%, 15%, and 20% CS with progressively severe shrinkage, and the minimum diameter (184.65 μm) was recorded at 20% CS. A slight diameter rebound was observed at 25% CS (200.86 μm), yet this value remained lower than that of the 0% group.

Collectively, 15% and 25% COS minimized tubular shrinkage in the COS system, while 5% CS produced the mildest shrinkage of seminiferous tubules among all CS groups.

## 4. Discussion

Testicular tissue cryopreservation is a critical strategy to preserve fertility in sexually mature males, yet its successful implementation faces substantial obstacles. Testicular tissue consists of diverse cell populations with distinct osmotic characteristics and cold tolerance. Intracellular ice crystal formation, osmotic imbalance, and oxidative stress during cryopreservation are among the major mechanisms responsible for reduced cell viability and disruption of tissue architecture [33,34]. Accordingly, supplementing cryoprotective media with high-efficiency, low-toxicity additives represents a core direction to optimize cryopreservation protocols. Functional carbohydrates, including COS and CS, exert cryoprotective effects through multiple pathways: they stabilize cellular membranes by interacting with phospholipid bilayers [35], regulate transmembrane water transport to mitigate osmotic stress, scavenge reactive oxygen species to alleviate oxidative damage [23,35], and reduce cryopreservation-induced apoptosis [19]. In recent years, functional carbohydrates have garnered extensive attention owing to their favorable biocompatibility and multi-layered protective mechanisms [36,37]. To our knowledge, this study applied COS and CS to slow freezing of rabbit testicular tissue and evaluated the effects of different treatments (0–25%) on post-thaw histomorphology and seminiferous tubule diameter. The results demonstrated that 20% COS and 15% CS provided the best histological preservation within their respective treatment series. The histomorphological score of the 20% COS group showed no significant difference from the fresh control group, whereas the 25% concentration group exhibited adverse alterations, including aggravated karyopyknosis and increased cellular vacuolation. These findings confirm the protective potential of functional carbohydrates for testicular cryopreservation and reveal their characteristic concentration-dependent effects.

The protective capacity of COS and CS appears to follow a biphasic concentration-dependent response under the present experimental conditions. A similar trend has been observed in studies of other saccharide-based cryoprotectants. Trehalose supplementation during cryopreservation of dairy goat testicular tissue has been reported to reduce apoptotic cell numbers and improve spermatogenesis-related gene expression, with 15% trehalose identified as the most effective concentration among those tested (5–25%) [19]. In the present study, histomorphological scores of COS groups increased from 4.63 ± 0.73 at 5% to 9.50 ± 0.32 at 20%, before declining to 7.37 ± 0.38 at 25%. For CS, the maximum score (8.18 ± 0.36) was observed at 15%, with diminished preservation at 20% and above. This concentration-dependent pattern appears comparable to that reported in a previous study [19]. The observed responses may be explained by the osmotic effects of non-permeable carbohydrates. At moderate concentrations, these compounds may elevate extracellular osmotic pressure, facilitate cellular dehydration and reducing the risk of intracellular ice formation. However, excessively high levels may generate a pronounced transmembrane osmotic gradient, triggering excessive cell shrinkage and irreversible damage. A concentration of 200 mM trehalose significantly improved germ cell recovery and proliferative capacity in porcine testicular cryopreservation, and this was the most effective concentration among those tested (50, 100, and 200 mM) [18]. Successful cryopreservation requires minimizing both osmotic injury caused by excessive cellular dehydration and damage associated with intracellular ice formation through appropriate control of cooling conditions and cryoprotectant exposure [1]. The elevated karyopyknosis and vacuolation observed in the 25% treatment groups suggest that osmotic stress may have exceeded cellular tolerance limits. The present findings indicate that COS possesses a wider effective concentration range than CS. COS exhibited peak protection at 20%, and although its protective efficacy declined at 25%, it remained superior to that observed at lower concentrations. In contrast, CS protection deteriorated more sharply beyond 15%. This difference might be related to the lower molecular weight of COS, which likely facilitates more uniform tissue distribution and reduces the risk of localized over-concentration. However, further studies are warranted to confirm this hypothesis.

COS exhibited superior overall cryoprotection relative to CS, with a histomorphological score of 9.50 ± 0.32 for the 20% COS group versus 8.18 ± 0.36 for the optimal CS group (15%). This difference may be related to their distinct molecular weights and associated physicochemical properties. CS is a high-molecular-weight polysaccharide (molecular weight > 100 kDa) with poor water solubility, which may reduce its diffusion into dense tissues. COS, by contrast, is a degraded derivative of CS with a degree of polymerization ranging from 2 to 20 and molecular weight below 10 kDa, exhibiting excellent water solubility and improved diffusibility compared to chitosan [22]. COS (degree of polymerization < 10) has been demonstrated to effectively alleviate oxidative injury in spermatogonia by reducing reactive oxygen species overproduction, mitigating endoplasmic reticulum stress, and restoring mitochondrial function [23]. This capacity to penetrate tissues and exert antioxidant activity is not observed with high-molecular-weight CS [23]. Furthermore, distinct cryosensitivity across cell types has been observed in testicular cryopreservation of multiple wild mammals, where round germ cells (spermatogonia, spermatocytes) generally withstand freezing better than elongated spermatids [38]. The small molecular structure of COS may allow for a more uniform distribution across cellular layers, potentially providing broader protection for different stages of spermatogenic cells. In contrast, high-molecular-weight CS primarily acts on the tissue surface by increasing extracellular matrix viscosity and forming a physical protective barrier, which may limit its access to spermatogonia located deeper within seminiferous tubules [20,21,23,24,39]. The favorable water solubility and low cytotoxicity of COS maintain robust biosafety even at relatively high concentrations [22]. Collectively, these properties may explain the superior protective effects of COS compared with CS at equivalent supplementation concentrations.

In addition to histomorphological scoring, this study assessed cryodamage to tissue architecture by measuring seminiferous tubule diameter. Seminiferous tubule diameters in all frozen groups were markedly smaller than those of the fresh control group, and the recovery trend of tubular diameter was inconsistent with histomorphological scores. This discrepancy suggests that the enlargement of seminiferous tubule diameter cannot be regarded as improved tissue protection; a larger diameter may result from cellular edema or a loose tissue structure instead of genuine restoration of structural integrity. Therefore, multiple indicators should be combined to evaluate cryoprotective efficacy. Histomorphological scores may reflect cellular and structural integrity, whereas seminiferous tubule diameter could be influenced by multiple biological and physical factors, including osmotic alterations, cellular edema, and tissue shrinkage. Joint analysis of both parameters may enable a more comprehensive assessment of protective performance.

Several important limitations of this study should be acknowledged. First, this research relies primarily on histological evaluation and seminiferous tubule diameter measurements. While histomorphological scoring is a valuable indicator of structural preservation, it does not directly demonstrate cell viability, apoptotic status, oxidative stress levels, or reproductive functionality after thawing. Second, samples were stored at −80 °C for one month rather than in liquid nitrogen, which is suitable for short-term preservation but may not ensure long-term stability for biobanking applications. Third, the use of adult rabbit testes, while justified for technical reasons as discussed above, means that the findings may not directly translate to prepubertal testicular tissue, which differs in cellular composition, extracellular matrix density, and cryosensitivity. Fourth, molecular-level indicators such as cell viability, apoptosis markers, oxidative stress parameters, and spermatogonial stem cell markers were not assessed. Consequently, the present findings should be interpreted as preliminary evidence of histological protection, rather than definitive proof of fertility preservation. Future studies should incorporate comprehensive functional assays to further validate the cryoprotective effects of COS and CS. These should include TUNEL staining to assess apoptosis, quantification of oxidative stress markers (e.g., ROS, MDA, SOD), immunodetection of spermatogonial biomarkers (e.g., PLZF, DDX4, or UCHL1), as well as in vitro tissue culture and xenotransplantation to evaluate the developmental and spermatogenic potential of cryopreserved testicular tissue. Such multi-parametric approaches would provide a more complete understanding of the biological protective mechanisms of COS and CS.

To our knowledge, this is the first study to apply COS and CS to the cryopreservation of rabbit testicular tissue, offering novel candidates for the development of functional polysaccharide cryoprotectants. The optimal supplemental concentrations of COS and CS were identified, and disparities in their protective performance were compared.

## 5. Conclusions

This study evaluated the cryoprotective efficacy of COS and CS at varying concentrations during the slow freezing of adult rabbit testicular tissues. The results demonstrate that these functional carbohydrates, when supplemented at appropriate concentrations, effectively preserve testicular microstructures, as assessed by histomorphological criteria, likely through mechanisms involving osmotic regulation and mitigation of ice crystal injury. Both COS and CS exhibited a typical biphasic concentration-response pattern, with optimal protection at 20% and 15%, respectively, while higher concentrations were associated with reduced efficacy.

COS consistently outperformed CS in terms of histological preservation under the present experimental conditions. This advantage may be attributed to the lower molecular weight and improved diffusibility of COS, which likely facilitates more uniform distribution within the tissue.

In conclusion, our findings identify COS and, to a lesser extent, CS as promising candidate additives for improving the histological quality of cryopreserved testicular tissue. While the present study provides a solid foundation for optimizing polysaccharide-based cryoprotectants, further investigations incorporating functional endpoints—such as cell viability, oxidative stress, apoptosis, and spermatogenic potential—will be valuable to fully establish the translational potential of this strategy for fertility preservation applications.

## Figures and Tables

**Table 1:** Cryoprotectant composition table.

Component (%)	Concentration (%)					
	1	2	3	4	5	6
Additive (COS or CS) % (*w*/*v*)	0	5	10	15	20	25
Dimethyl sulfoxide % (*v*/*v*)	10	10	10	10	10	10
Fetal bovine serum % (*v*/*v*)	20	20	20	20	20	20
Penicillin-streptomycin % (*v*/*v*)	5	5	5	5	5	5
DMEM % (*v*/*v*)	65	60	55	50	45	40

COS and CS groups were prepared with identical compositions, except that the additive was COS for the COS groups and CS for the CS group. Concentrations of additives are expressed as % (*w*/*v*), and all other components as % (*v*/*v*). COS, chitosan oligosaccharide; CS, chitosan.

**Table 2:** Effects of different COS concentrations on histomorphological scores of rabbit testicular tissues (mean ± SD).

Group	Cell Swelling	Cell Loss	Basement Membrane Rupture	Basement Membrane Shrinkage	Tubular Structure	Total Score
Fresh	1.97 ± 0.08 ^a^	1.93 ± 0.16 ^a^	1.97 ± 0.08 ^a^	1.85 ± 0.19 ^a^	1.93 ± 0.16 ^a^	9.65 ± 0.67 ^a^
0% COS	0.48 ± 0.12 ^d^	0.13 ± 0.12 ^e^	1.00 ± 0.21 ^c^	0.47 ± 0.14 ^d^	0.50 ± 0.14 ^d^	2.58 ± 0.69 ^f^
5% COS	1.03 ± 0.16 ^c^	0.53 ± 0.16 ^d^	1.52 ± 0.15 ^b^	0.53 ± 0.12 ^d^	1.02 ± 0.15 ^c^	4.63 ± 0.73 ^e^
10% COS	1.13 ± 0.12 ^c^	1.05 ± 0.10 ^c^	1.55 ± 0.10 ^b^	1.02 ± 0.15 ^c^	1.12 ± 0.12 ^c^	5.87 ± 0.56 ^d^
15% COS	1.55 ± 0.10 ^b^	1.45 ± 0.10 ^b^	1.98 ± 0.04 ^a^	1.53 ± 0.10 ^b^	1.57 ± 0.08 ^b^	8.08 ± 0.40 ^b^
20% COS	1.93 ± 0.08 ^a^	1.90 ± 0.09 ^a^	2.00 ± 0.00 ^a^	1.87 ± 0.10 ^a^	1.80 ± 0.18 ^a^	9.50 ± 0.32 ^a^
25% COS	1.48 ± 0.08 ^b^	1.93 ± 0.08 ^a^	1.97 ± 0.05 ^a^	0.98 ± 0.12 ^c^	1.00 ± 0.09 ^c^	7.37 ± 0.38 ^c^

**^a,b,c,d,e,f^** Different superscript letters within the same column indicate significant differences among all groups (including the fresh control, 0% COS, and 5–25% COS treatment groups) as determined by one-way ANOVA followed by Duncan’s multiple range test (*p* < 0.05). The total score represents the sum of the five individual indicators. The scoring criteria were adapted from previously established histological evaluation systems for cryopreserved testicular tissues [30,31]. For each indicator, a score of 2 represents normal or near-normal morphology with no or minimal alterations (<20% of cells or tubules affected), a score of 1 indicates moderate damage (20–50% of cells or tubules affected), and a score of 0 denotes severe damage (>50% of cells or tubules affected). Specifically, for cell swelling, a score of 2 indicates no or minimal swelling, 1 indicates moderate swelling, and 0 indicates severe swelling involving the majority of cells. For cell loss, a score of 2 corresponds to no or minimal detachment of epithelial cells, 1 indicates moderate cell loss, and 0 indicates extensive cell loss from the seminiferous epithelium. For basement membrane rupture, a score of 2 indicates an intact basement membrane, 1 indicates partial rupture, and 0 indicates severe rupture or discontinuity. For basement membrane shrinkage, a score of 2 indicates no or minimal shrinkage, 1 indicates moderate shrinkage, and 0 indicates severe shrinkage affecting the majority of tubules. For seminiferous tubule structural integrity, a score of 2 indicates well-organized and intact tubular architecture, 1 indicates moderate disorganization with partial loss of architecture, and 0 indicates complete loss of tubular architecture with severe disorganization. The percentage values refer to the proportion of seminiferous tubules or cells affected within each randomly selected microscopic field. Three observers performed the scoring independently, and the final score for each indicator was calculated as the mean of the three readings. COS, chitosan oligosaccharide; CS, chitosan.

**Table 3:** Effects of different CS concentrations on histomorphological scores of rabbit testicular tissues (mean ± SD).

Group	Cell Swelling	Cell Loss	Basement Membrane Rupture	Basement Membrane Shrinkage	Tubular Structure	Total Score
Fresh	1.85 ± 0.05 ^a^	1.82 ± 0.10 ^a^	1.90 ± 0.09 ^a^	1.73 ± 0.08 ^a^	1.83 ± 0.08 ^a^	9.13 ± 0.39 ^a^
0% CS	0.37 ± 0.08 ^f^	0.28 ± 0.08 ^e^	0.42 ± 0.12 ^e^	0.37 ± 0.08 ^e^	0.37 ± 0.08 ^e^	1.80 ± 0.42 ^e^
5% CS	0.75 ± 0.10 ^e^	0.67 ± 0.12 ^d^	1.03 ± 0.12 ^d^	0.75 ± 0.10 ^d^	0.88 ± 0.15 ^d^	4.08 ± 0.60 ^d^
10% CS	1.15 ± 0.10 ^d^	1.15 ± 0.10 ^c^	1.38 ± 0.08 ^c^	1.18 ± 0.08 ^c^	1.28 ± 0.08 ^c^	6.15 ± 0.41 ^c^
15% CS	1.58 ± 0.08 ^b^	1.55 ± 0.10 ^b^	1.75 ± 0.05 ^b^	1.62 ± 0.08 ^b^	1.68 ± 0.08 ^b^	8.18 ± 0.36 ^b^
20% CS	1.28 ± 0.08 ^c^	1.18 ± 0.08 ^c^	1.48 ± 0.08 ^c^	1.18 ± 0.08 ^c^	1.28 ± 0.08 ^c^	6.42 ± 0.38 ^c^
25% CS	0.75 ± 0.10 ^e^	0.65 ± 0.10 ^d^	0.95 ± 0.10 ^d^	0.75 ± 0.10 ^d^	0.85 ± 0.10 ^d^	3.95 ± 0.52 ^d^

**^a,b,c,d,e,f^** Different superscript letters within the same column indicate significant differences among all groups (including the fresh control, 0% CS, and 5–25% CS treatment groups) as determined by one-way ANOVA followed by Duncan’s multiple range test (*p* < 0.05). The total score represents the sum of the five individual indicators. Related scoring rules are the same as Table 2. COS, chitosan oligosaccharide; CS, chitosan.

## Data Availability

The original contributions presented in this study are included in the article. Further inquiries can be directed to the corresponding authors.
